# An Improved Quantitative Analysis Method for Plant Cortical Microtubules

**DOI:** 10.1155/2014/637183

**Published:** 2014-03-10

**Authors:** Yi Lu, Chenyang Huang, Jia Wang, Peng Shang

**Affiliations:** ^1^School of Life Sciences, Northwestern Polytechnical University, 127 Youyi xilu, Xi'an, Shaanxi 710072, China; ^2^School of Science, Northwestern Polytechnical University, 127 Youyi xilu, Xi'an, Shaanxi 710072, China

## Abstract

The arrangement of plant cortical microtubules can reflect the physiological state of cells. However, little attention has been paid to the image quantitative analysis of plant cortical microtubules so far. In this paper, Bidimensional Empirical Mode Decomposition (BEMD) algorithm was applied in the image preprocessing of the original microtubule image. And then Intrinsic Mode Function 1 (IMF1) image obtained by decomposition was selected to do the texture analysis based on Grey-Level Cooccurrence Matrix (GLCM) algorithm. Meanwhile, in order to further verify its reliability, the proposed texture analysis method was utilized to distinguish different images of Arabidopsis microtubules. The results showed that the effect of BEMD algorithm on edge preserving accompanied with noise reduction was positive, and the geometrical characteristic of the texture was obvious. Four texture parameters extracted by GLCM perfectly reflected the different arrangements between the two images of cortical microtubules. In summary, the results indicate that this method is feasible and effective for the image quantitative analysis of plant cortical microtubules. It not only provides a new quantitative approach for the comprehensive study of the role played by microtubules in cell life activities but also supplies references for other similar studies.

## 1. Introduction 

Microtubules exist in all eukaryotic cells. They are long tubular organelles assembled with *αβ*-tubulin dimers. Microtubules are involved in almost all basic biological processes, such as cell division [1], cell morphogenesis [2], and signal transmission and transduction [3]. In plant cell, the arrangement of the cortical microtubules is closely related to cell growth, by which the deposition direction of cell microfibrils is controlled [4]. For the reason that the polymerization and depolymerization of microtubules are a highly dynamic process, small changes in the external environment (such as mechanical stimuli [5, 6], hormone [5], and light [7]) may lead to alterations in the arrangement of microtubules. Generally speaking, the fluorescence microscopy and filming techniques are used to observe and describe the morphology of microtubules. However, there are many drawbacks in the traditional method which makes use of descriptive language determination. Thus, in order to understand the function of plant microtubules in response to the changes of environment, it is necessary to define the changes by precise and efficient quantitative determination.

As a significant appearance characteristic, texture can reflect the image properties of cytoskeleton, owing to which texture analysis is frequently applied to describe the microtubules. Texture analysis refers to texture parameters extracted through some image processing techniques to describe texture quantitatively [8]. Grey-Level Cooccurrence Matrix (GLCM) algorithm, based on the second-order combination of the conditional probability density of the image, is more efficient in characterizing textures [9]. As for GLCM, the complexity of texture can be reflected accurately by the image's direction, interval, and changes in amplitude and speed information through calculating the correlation between a certain distance and the direction of the image grayscale.

However, during the image generation, transmission, or transformation process, due to the influence of various objective factors, the reducing of image quality frequently happens and leads to loss or less prominent of image texture feature. Therefore, it is essential to firstly make the image preprocessing before analyzing.

The Empirical Mode Deposition (EMD) approach was introduced for the complex signals smooth processing by Huang et al. in 1998 [10] and also known as Hilbert-Huang transform. It is mainly applied in multiresolution analysis of one-dimensional time sequence signal. This method decomposes the signal into a series of intrinsic mode functions (IMF) and a residue. These intrinsic mode functions satisfy the orthogonality and completeness, and when combined with the residue they can achieve perfect reconstruction of the original signal. In 2003, the EMD algorithm was extended to the 2-dimensional scaling and then applied in the image decomposition by Nunes et al. [11]. Called as Bidimensional Empirical Mode Decomposition (BEMD), it has been successfully used in geological exploration [12], ocean exploration [13], biomedical [14], and other signal denoising and fault diagnoses research areas [15].

Here we propose to combine BEMD with GLCM algorithm to analyze the texture by means of the microscope image of the Arabidopsis cortical microtubules. The method can be used to show different status of microtubule arrangements that have been observed by the microscope. These findings provide a more effective and convenient method for the quantitative analysis of plant microtubules and for the better understanding of the underlying mechanisms of microtubules in the biological process.

## 2. Materials and Methods

### 2.1. Plant Material and Growth Conditions

Transgenic Arabidopsis* thaliana* GFP-TUA6 (Columbia ecotype) was used throughout the experiments. The Arabidopsis seeds were sterilized with 75% alcohol for 30 s and 10% NaOCl solution for 10 mins, and then they were washed 5-6 times with double distil H_2_O. Surface-sterilized seeds were sown on the 0.5x MS medium (1% w/v sucrose, 0.8% w/v agar, pH 5.8) in a line and grown in the illumination incubator (the intensity of illumination was 120 *μ*mol/m^2^/s and the photoperiod was 16 h light/8 h dark) at 25°C until they were able to be observed.

### 2.2. Image Acquisition

The 4-day-old Arabidopsis seedlings were removed from culture medium quickly. We put the seedlings on glass slide, immersed them in distil water, and covered them with a coverslip (24 × 60 mm) carefully. Living image acquisition was performed on Leica confocal microscope by Leica ×63 N.A. = 1.4 oil immersion objective. GFP was excited at 488 nm and detected at 500–530 nm. The pixel of the acquired image was 1024 × 1024. All of the images were saved in TIFF format.

### 2.3. The Bidimensional Empirical Mode Decomposition Analysis

**Figure 1:**
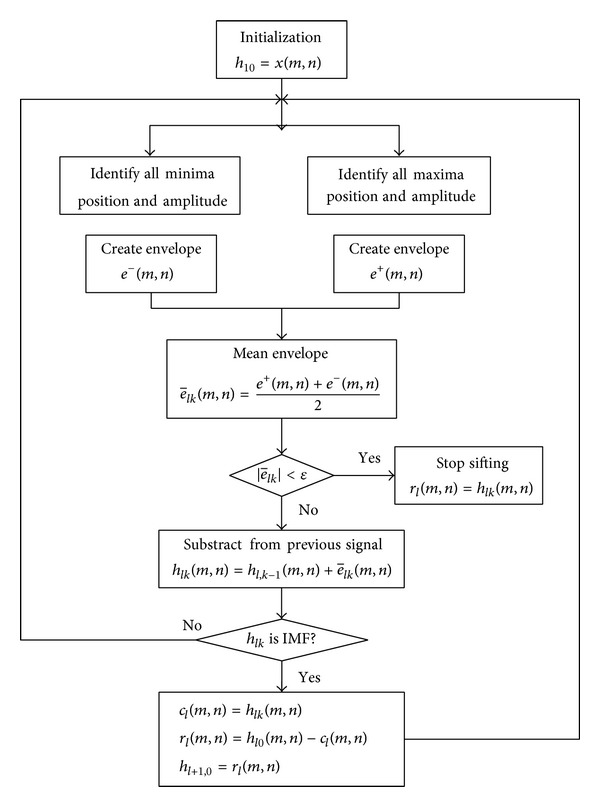
Sifting process of BEMD.

BEMD algorithm which referred to the method proposed by Nunes et al. was written and performed through Matlab 7.0 software. Conceptually similar to the one-dimensional EMD screening process except for the curve fitting of the maxima and minima envelope, the arithmetic of the BEMD algorithm is shown in Figure 1.

The original signal is *x*(*m*, *n*), and the two-dimensional sifting process is illustrated in Figure 1. The first index *l* = 1,…, *L* means the IMF number, the second index *k* = 1,…, *K* means the iteration number, and *m* and *n* represent the two spatial dimensions. After the sifting process, IMFs of the signal are put into *c*(*m*, *n*) and the residual into *r*(*m*, *n*). *h*_*lk*_ are intermediate variables. The original signal can be expressed as

(1) $$\begin{array}{c} x\left(m,n\right)=\overset{L}{\underset{l=1}{\sum }}c_{l}\left(m,n\right)+r_{L}\left(m,n\right). \end{array}$$

### 2.4. GLCM Analysis

GLCM algorithm was proposed by Haralick et al. in early 70s [16]. It is built in the conditions probability density function of the image. By calculating the probability of the two pixels in a specific direction and distance, the comprehensive information on the direction, distance, and the magnitude of image changes can be reflected.

In this paper, image texture analysis based on GLCM was performed through Matlab 7.0 software. Here, based on Haralick et al. [16] and the results of a preliminary study by our group [17], we set *d* = 4, and then extracted five texture parameters: angular second moment, entropy, inverse different moment, variance, and contrast. Thereinto, the first 4 parameters were selected to quantitatively distinguish the different morphology of cortical microtubules. The contrast value was used as a criterion in the effect of BEMD processing evaluation.

(1) Angular second moment (ASM):

(2) $$\begin{array}{c} \text{ASM}=\overset{G-1}{\underset{i=0}{\sum }}\;\overset{G-1}{\underset{j=0}{\sum }}{\left\{P\left(i,j\right)\right\}}^{2}. \end{array}$$

(2) Entropy:

(3) $$\begin{array}{c} \text{Entropy}=\overset{G-1}{\underset{i=0}{\sum }}\;\overset{G-1}{\underset{j=0}{\sum }}P\left(i,j\right)\times \log\left(P\left(i,j\right)\right). \end{array}$$

(3) Inverse different moment (IDM):

(4) $$\begin{array}{c} \text{IDM}=\overset{G-1}{\underset{i=0}{\sum }}\;\overset{G-1}{\underset{j=0}{\sum }}\frac{1}{1+{\left(i-j\right)}^{2}}P\left(i,j\right). \end{array}$$

(4) Variance:

(5) $$\begin{array}{c} \text{Variance}=\overset{G-1}{\underset{i=0}{\sum }}\;\overset{G-1}{\underset{j=0}{\sum }}{\left(i-\mu \right)}^{2}P\left(i,j\right). \end{array}$$

(5) Contrast:

(6) $$\begin{array}{c} \text{Contrast}=\overset{G-1}{\underset{n=0}{\sum }}n^{2}\left\{\overset{G}{\underset{i=1}{\sum }}\overset{G}{\underset{j=1}{\sum }}P\left(i,j\right)\right\},\;\left|i-j\right|=n. \end{array}$$

### 2.5. Statistical Analysis

GraphPad Prism 5.0 softwarewas used to do *t*-test analysis and to draw line charts and bar charts. All experiments were independently repeated at least three times. Results were reported as mean ± standard deviation (SD). *P* value <0.05 was taken as statistical significance.

## 3. Results

### 3.1. Image Enhancement Based on BEMD

**Figure 2:**
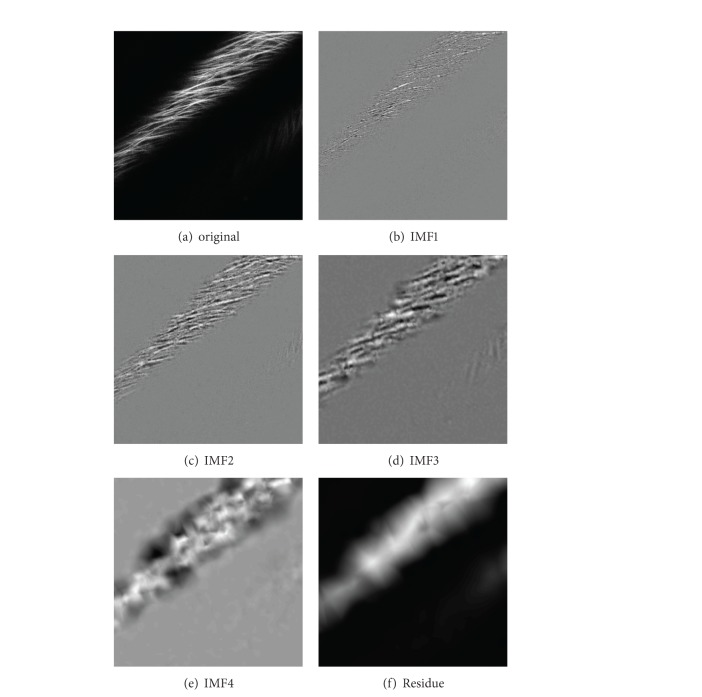
The image of plant microtubules decomposed by the BEMD algorithm.

The BEMD algorithm was applied in this paper to improve the image quality of Arabidopsis microtubules. It can be seen from Figure 2 that the grayscale original image is decomposed gradually to four IMFs (Intrinsic Mode Functions) images and a residue image depending on different frequencies of signals. The IMF images reflect the details of the original image from different scales and the residue image reflects the trend information. The order of clarity of these images is IMF1 > IMF2 > IMF3 > IMF4 > residue image.

**Figure 3:**
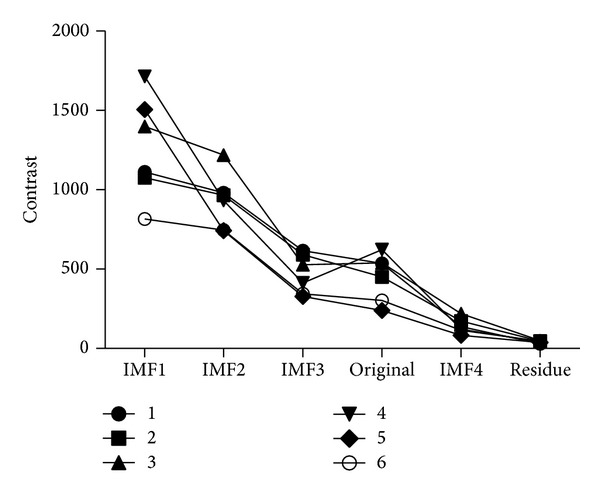
Contrast comparison of IMFs, residue and original images.

Texture contrast extracted by GLCM reflects the sharpness of the image. It is recognized that the greater the value of contrast is, the clearer the image texture is, and vice versa. By comparing the original image with the four IMF images and the residue image, (Figure 3), we found that the order of the contrast value of these images is IMF1 > IMF2 > IMF3 > IMF4 > residue image. The contrast values of the original image and IMF3 image are relatively close, which indicates an insignificant difference of the clarity of these two images (Figure 3).

### 3.2. Image Texture Analysis of Microtubules Based on GLCM

After image enhancement, the IMF1 image (Figure 2(b)) was used to make texture analysis and extract the texture features in four directions by GLCM. We found that the range of angular second moment, entropy, inverse difference moment, and variance values in four directions are relatively stable (Table 1). The results indicate that the four texture features have good rotation invariance, which can effectively reflect some features of the original image.

### 3.3. Application Verification of the Texture Analysis Method

**Figure 4:**
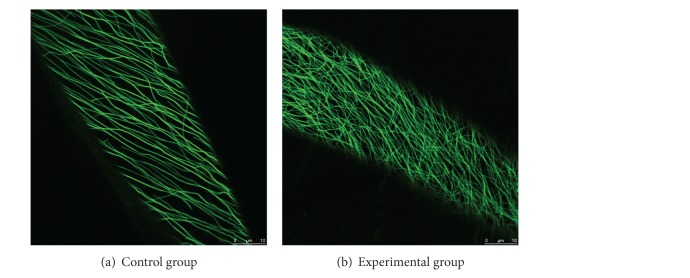
Two different arrangement images of plant microtubules.

**Figure 5:**
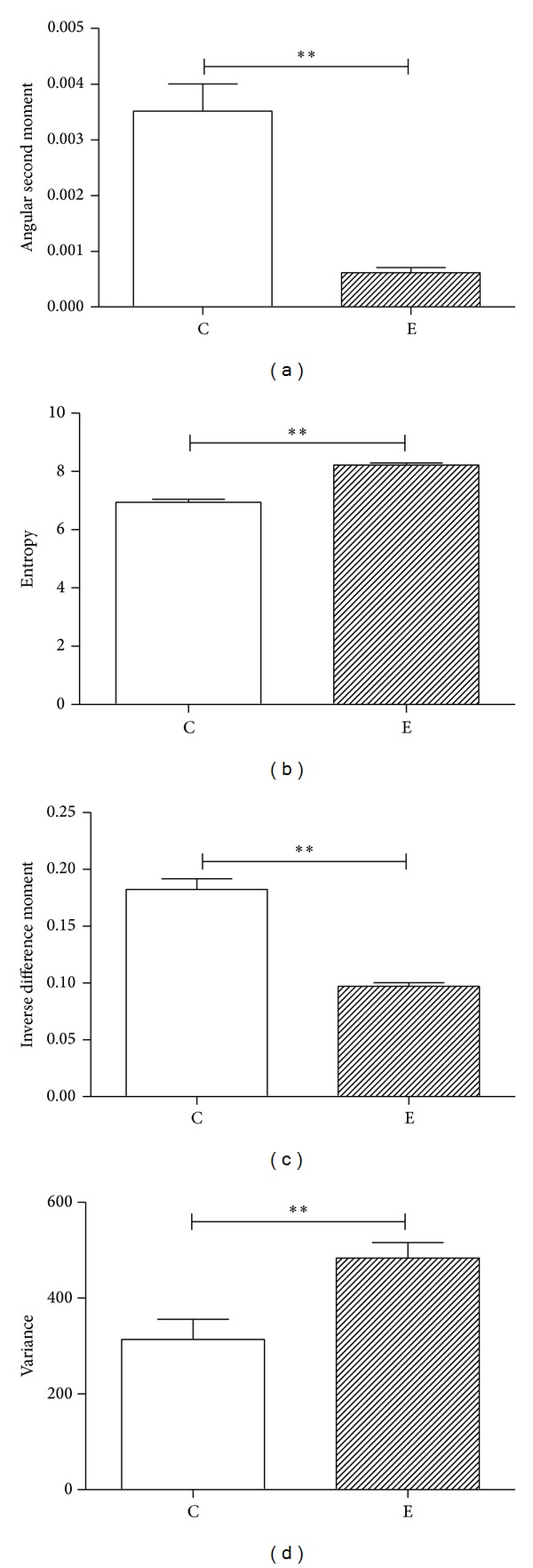
*t*-test analysis of four texture features of plant microtubules between different images. (a) *t*-test analysis of angular second moment; (b) *t*-test analysis of entropy; (c) *t* -test analysis of inverse difference moment; (d) *t*-test analysis of variance. C: control group; E: experimental group. **P < 0.01.

To further verify the application of the texture analysis method in this paper, two different kinds of fluorescence images of Arabidopsis cortical microtubules were selected to make comparison (Figure 4). Statistical analysis (Figure 5) shows that, compared with the control group, there are significant differences in the four image texture features (angular second moment, entropy, inverse different moment and variance) of the experimental group (treated by simulated microgravity), which is consistent with the reflected information of fluorescence images.

Angular second moment reflects the uniformity or smoothness of the image. The more detail is shown in the image, the higher value of angular second moment is calculated. On the contrary, when the image intensity distribution is greatly uneven showing rough surface characteristics, the value of angular second moment will be small. Entropy indicates texture complexity. The higher the complexity of the image is, the greater the entropy value is. Inverse difference moment reflects the degree of texture regularity. The smaller the value of inverse difference moment is, the more difficult the description of texture is, because the texture is disorganized. Conversely, the higher the value of inverse difference moment is, the easier the description of texture is, because the texture is regular. Variance is another important feature which reflects the texture periodicity. The greater the variance value is, the faster the changing frequency of texture periodicity is, and vice versa. In summary, it can be seen that these four parameters perfectly represent the most of the image information, indicating that the texture analysis used here can reflect visual messages and can distinguish different textures.

## 4. Discussion

Texture analysis, an important research area in digital image processing, has a very broad application prospects. Currently, the texture analysis method can be divided into four categories: statistics, structure method, modelling, and transformation method based on spectrum analysis. The method based on GLCM is one of the most classic and famous statistical methods. It plays an important role in the texture analysis area. Since last century, the GLCM algorithm have been widely used in texture classification and texture segmentation fields as well as remote sensing and biomedical image analysis and many other application fields. Weszka et al. [18] have compared the texture analysis methods of Fourier spectrum, autocorrelation function, and GLCM and have recognized that the classification performance of GLCM is better than that of the Fourier spectrum and spatial frequency analysis method. The similar conclusions of performance comparison studies were made by Conners and Harlow [19].

In the biomedical field, researchers mainly apply image analysis to assist the doctor for diagnosis [20]. For example, by analysing the image texture features of MRI, micro-CT, X-rays, and pathological sections, researchers can distinguish the cancerous cells from the normal cells, and the abnormal tissues from the normal tissues, thereby helping doctors to diagnose whether a patient has a disease. In recent years, with the continuous development of the microscopic imaging technology, more and more researchers introduce texture analysis method to cell biology research. Shamir et al. [21] have proposed to use the image texture entropy as an objective measurement which can reflect the structural deterioration of the C.* elegans* muscle tissues during aging. Lichtenstein et al. [22] have applied the FiberScroe algorithm in quantitative analysis of the cytoskeleton of Swiss 3T3 fibroblasts, which marks the beginning of the cytoskeleton analysis from qualitative to quantitative. Subsequently, on this basis, Shah et al. [23] have optimized and improved the speed of operation providing with view interface; Qian et al. [24] have used the fractal dimension to measure the arrangement of actin cytoskeleton in MC3T3-E1 cells to reveal the influence of simulated microgravity on the cells.

All of these studies have focused on animal cell research, and, compared with animal's microtubule, the plant microtubule has its own characteristics. Therefore, the texture analysis based on GLCM was used for quantitative analysis of plant cortical microtubule in this paper. However, in the biological experiments, because of the influence of many objective factors, the quality of the acquired image or signal to noise ratio (SNR) is low, which will also affect the results of analysis and calculation and reduce the accuracy of the image classification. It is necessary to make image enhancement. Image enhancement is a kind of processing method which caters to specific needs such as highlighting some of the information and weakening or removing some undesirable information in an image. Its main purpose is to improve the image which should be processed and to improve the appearance of the original image, so that the image is more suitable for human's visual judgment or analysis by machine.

In this paper, the BEMD algorithm is applied in the original image enhancement, and the effect is remarkable. The number of the IMF image is smaller; the image is clearer, which is suitable for the subsequent analysis. This is because the decomposition process of BEMD is a continuous extraction process of high frequency component. Compared with Fourier, wavelet transform, and other image analysis methods, BEMD is completely data-driven with excellent local analysis capabilities. It is demonstrated that BEMD is an adaptive, completely unsupervised method which is more efficient in nonlinear and nonstationary signal analysis and processing. The comparison between the two different arrangements of microtubule image demonstrates that the use of four parameters can perfectly represent the alteration of cortical microtubule array and may reveal the functional changes. It also indicates that the application of this texture analysis method is feasible and effective. However, there is no absolutely perfect method and everyone has its own drawbacks and application scope. The application of the proposed method has its limitations. Firstly, the principle of the BEMD algorithm shows that the obtained image was decomposed according to the frequency of signal. If the noise signal of the image which needs to be processed is the highest frequency, the image information of IMF1 is complete noise. Thus, whether or not IMF1 image is used depending on the main frequency band of the target information exists in original image. In this paper, IMF1 is adopted to analyze because the useful information signal of analyzed image is in the highest frequency, and this is true in most biological experiment. If the information of collected object image is almost masked by noises, which indicates that the experiment itself is failure and untrusted, and it is not necessary to make quantitative analysis. Therefore, the purpose of this noise reduction method proposed in this paper is to highlight the target information, minimize noise interference, and improve the accuracy of subsequent GLCM analysis. Moreover, the calculation of GLCM algorithm is massive, and therefore it should determine the appropriate *d* value and screen texture parameters before texture analysis by using GLCM in accordance with the actual situation in image processing, which can reduce the redundant information and workload.

## 5. Conclusion

In summary, a feasible and effective texture analysis method is established based on BEMD and GLCM algorithm for plant cortical microtubule quantitative analysis. By this method, the artificial subjective factors can be avoided, so that the evaluation is objective and quantitative, and the accuracy of analysis is improved. Furthermore, it provides references to other related research areas.

## Figures and Tables

**Table 1:** The texture features extraction based on GLCM algorithm.

Direction	Angular second moment	Entropy	Inverse difference moment	Variance
0°	0.002	6.5	0.065	691.534
45°	0.002	6.509	0.059	681.668
90°	0.002	6.523	0.058	690.952
135°	0.002	6.55	0.056	702.012

## Abbreviations

BEMD: Bidimensional Empirical Mode Decomposition
CT: Computer tomography
EMD: Empirical Mode Deposition
GFP: Green fluorescent protein
GLCM: Grey-Level Cooccurrence Matrix
IMF: Intrinsic Mode Function
MRI: Magnetic resonance imaging
TUA: *α*-TUBULIN
SNR: Signal to noise ratio
TIFF: Tagged image file format.
